# A multimodal spatio-temporal graph neural network framework for fatigue detection in tennis serving

**DOI:** 10.3389/fbioe.2026.1828589

**Published:** 2026-06-09

**Authors:** Ye Yuan, Lu Wang, Hui Jia, Junfeng Jiao

**Affiliations:** 1 College of Physical Education, Xuzhou University of Technology, Xuzhou, China; 2 Physical Education Department, Harbin Institute of Technology, Harbin, China; 3 Department of Marine-Sports, Pukyong National University, Busan, Republic of Korea; 4 Physical Education Department of Anhui Sanlian University, Hefei, China

**Keywords:** fatigue detection, graph neural networks, kinetic chain, sensor fusion, sports medicine, tennis biomechanics, wearable sensors

## Abstract

**Background:**

The tennis serve is a complex, high-velocity motion dependent on the efficient transfer of energy through the kinetic chain, from the lower extremities to the racquet. Fatigue-induced alterations in this chain are primary precursors to overuse injuries, such as rotator cuff tendinopathy and elbow medial collateral ligament stress. However, current wearable monitoring solutions predominantly rely on unimodal kinematic data, failing to capture the neuromuscular compensatory mechanisms that precede mechanical performance degradation.

**Methods:**

This study introduces a novel multimodal framework integrating inertial measurement units (IMUs) and surface electromyography (sEMG) with a Spatio-Temporal Graph Convolutional Network (ST-GCN). We recruited 15 high-performance tennis players to perform a standardized specific endurance protocol (serving to exhaustion). A graph topology representing the human skeletal structure was constructed to model spatial dependencies, while sEMG signals were fused as node attributes to capture neural drive intensity. Statistical significance was assessed using paired ***t***-tests with Bonferroni correction, and model performance differences were evaluated by one-way repeated measures ANOVA with post-hoc pairwise comparisons.

**Results:**

The proposed ST-GCN framework achieved a fatigue state classification accuracy of 95.2% (F1-score: 0.94), significantly outperforming traditional Long Short-Term Memory (LSTM) (88.1%) and Convolutional Neural Network (CNN) architectures (85.6%), as well as a Spatial-Temporal Transformer network (ST-TR) (93.1%) (***p*** < 0.001 for all pairwise comparisons). Biomechanical analysis revealed a significant “neuromechanical lag” (p < 0.01) and a compensatory pattern characterized by reduced knee flexion (−12.4°) during the fatigued state, accompanied by a non-significant upward trend in shoulder internal rotation velocity (+8.5%, p = 0.012).

**Conclusion:**

By decoding the hidden dependencies within the kinetic chain, this system provides a computationally efficient fatigue detection framework with a model inference latency of 8.4 ms, constituting a technically feasible step toward on-court deployment and offering a potential paradigm for smart sports engineering and precision rehabilitation.

## 1 Introduction

Previous studies have provided important methodological foundations for this work. Multi-sensor fusion and wearable-sensor-based activity recognition have been widely reviewed and applied in human movement analysis (Aguileta et al., 2019; Taborri et al., 2016; Chen et al., 2012; Chen et al., 2021; Wu et al., 2021). Deep learning optimization and representation-learning methods, including Adam optimization and t-SNE visualization, have also supported the development and interpretation of sensor-based models (Kingma and Ba, 2015; van der Maaten and Hinton, 2008). In sports biomechanics and injury-related movement analysis, previous work has examined shoulder function, tennis performance, and fatigue-related motion characteristics (Roetert et al., 2009; Andrews et al., 2009; Martin et al., 2011; Martin et al., 2016; Phinyomark et al., 2018; Dang et al., 2020; Shi et al., 2019).

The tennis serve is widely regarded as the most dominant and physically demanding shot in modern tennis, accounting for approximately 45%–60% of all strokes played during a match. Biomechanically, the serve relies on the “kinetic chain” principle—a sequential activation of body segments that generates, sums, and transfers energy from the ground reaction forces through the legs, trunk, and upper arm to the racquet. Efficiency in this chain minimizes the load on individual joints while maximizing ball velocity. However, the repetitive and high-intensity nature of serving makes players highly susceptible to neuromuscular fatigue. Unlike acute fatigue, which manifests as an immediate decline in performance, sub-maximal fatigue in tennis often induces subtle biomechanical compensations. Players may unconsciously alter their coordination strategies to maintain serve velocity, leading to the “catch-up” phenomenon where distal segments (e.g., the shoulder or elbow) experience excessive loading to compensate for reduced energy generation in proximal segments (e.g., the legs). Epidemiological studies indicate that chronic overuse injuries resulting from such maladaptive patterns account for over 50% of time-loss injuries in elite tennis players (Pluim et al., 2006;Chung and Lark, 2017). Therefore, the continuous, objective monitoring of the kinetic chain status is critical for injury prevention and training load management.

Traditionally, the gold standard for analyzing tennis biomechanics involves optical motion capture systems (e.g., Vicon, Qualisys) combined with force plates. While these systems offer millimeter-level precision, their confinement to laboratory settings, high cost, and complex setup requirements render them impractical for daily on-court monitoring. To address this, wearable technology has emerged as a ubiquitous alternative. Recent advancements in Micro-Electro-Mechanical Systems (MEMS) have enabled the deployment of lightweight Inertial Measurement Units (IMUs) to track segment kinematics in ecological environments. Numerous studies have successfully utilized IMUs to measure serve velocity, classify stroke types, and estimate joint angles (Rigozzi et al., 2023; Whiteside et al., 2013). However, a significant limitation of existing IMU-based approaches is their unimodal focus on kinematics (motion) while neglecting kinetics (force/muscle activity). Fatigue is fundamentally a neuromuscular phenomenon; changes in neural drive often precede observable kinematic deviations. Relying solely on kinematic data may result in a “lagged” detection of fatigue, identifying the risk only after the movement pattern has already significantly deteriorated.

To capture the physiological precursors of fatigue, surface electromyography (sEMG) provides a direct window into the neural control of movement. Parameters such as the Mean Power Frequency (MPF) and the Root Mean Square (RMS) of the sEMG signal are established indicators of local muscle fatigue. Yet, sEMG signals are highly stochastic and susceptible to motion artifacts, especially during high-velocity movements like the tennis serve. Furthermore, current literature largely treats kinematic and EMG data as separate domains. Few studies have attempted to fuse these modalities to investigate the neuromechanical coupling—that is, how the timing and intensity of muscle activation map onto the resulting segmental acceleration (Chung et al., 2019). The integration of these heterogeneous data streams (time-series kinematic data and spectral muscle activity data) poses a significant challenge for traditional signal processing and machine learning algorithms.

In the domain of human activity recognition and sports analytics, Deep Learning (DL) has become the state-of-the-art methodology. Conventional architectures such as Convolutional Neural Networks (CNNs) and Recurrent Neural Networks (RNNs, including LSTMs) have been widely applied to wearable sensor data. While effective for temporal feature extraction, these models fundamentally treat the human body as a sequence of independent sensor readings or a grid-like image, ignoring the natural topological connectivity of the human skeleton. The human body is articulated; the movement of the wrist is physically constrained by the elbow and shoulder, and functionally linked to the contralateral leg through the kinetic chain. Neglecting these spatial dependencies limits the model’s ability to capture the subtle coordination changes characteristic of kinetic chain breakdown. Recently, Graph Neural Networks (GNNs), specifically Spatio-Temporal Graph Convolutional Networks (ST-GCN), have demonstrated superior performance in skeleton-based action recognition by modeling the human body as a graph structure, where joints are nodes and bones are edges (Yan et al., 2018; Wang et al., 2019). Attention-based architectures, such as the Spatial-Temporal Transformer (ST-TR), have further advanced this field by replacing fixed graph convolutions with dynamic self-attention mechanisms (Plizzari et al., 2021). This topology-aware approach is theoretically ideal for analyzing the kinetic chain but has not yet been rigorously applied to multimodal fatigue detection in racquet sports.

This paper proposes a novel Multimodal Wearable Framework utilizing a Spatio-Temporal Graph Neural Network to quantify fatigue-induced alterations in the tennis serve. Unlike previous studies that isolate specific joints or rely on single-sensor modalities, our approach integrates whole-body kinematics from a distributed IMU network with muscle activation data from wireless sEMG sensors. We hypothesize that (1) the fusion of neural and mechanical data will significantly enhance fatigue detection accuracy compared to unimodal baselines; and (2) the interpretable features learned by the GNN will reveal specific compensatory mechanisms—specifically, a breakdown in leg drive necessitating increased shoulder internal rotation—that standard “black-box” models fail to identify. The contributions of this work include the development of a synchronized neuro-mechanical sensor network, the first application of ST-GCN for kinetic chain fatigue analysis, and the identification of quantifiable biomarkers for shoulder injury risk in tennis.

## 2 Materials and methods

### 2.1 Participants and ethics statement

Fifteen semi-professional and high-performance collegiate tennis players (10 males, 5 females; age: 21.4 ± 2.3 years; height: 178.6 ± 6.2 cm; weight: 74.5 ± 8.1 kg) were recruited for this study. The inclusion criteria were: (1) an International Tennis Number (ITN) of 4 or better, indicating an advanced level of play; (2) a minimum of 8 years of competitive playing experience; and (3) no history of musculoskeletal injuries to the upper or lower extremities in the 6 months preceding the experiment. All participants were right-handed. The study protocol was approved by the University Institutional Review Board (IRB) and adhered to the Declaration of Helsinki. Written informed consent was obtained from all subjects prior to data collection.

### 2.2 The multimodal sensing system

**FIGURE 1:**
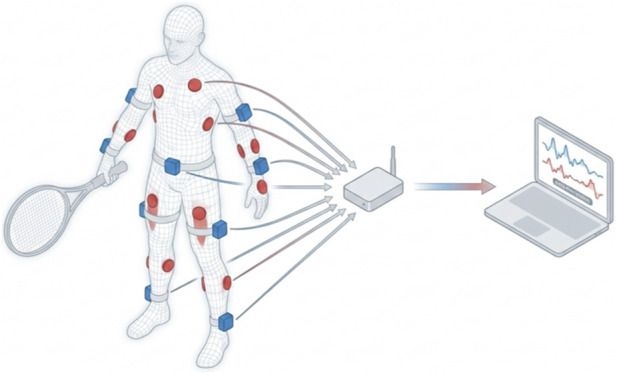
The multimodal wearable sensing system.

To capture the comprehensive dynamics of the kinetic chain, we designed a synchronized heterogeneous sensor network consisting of IMUs for kinematics and sEMG sensors for muscle activity. The overall wearable sensing system is shown in Figure 1.

#### 2.2.1 Inertial measurement units (IMUs)

We utilized eight wireless 9-axis IMUs (Delsys Trigno Avanti, Delsys Inc., Natick, MA, USA; sampling rate: 148 Hz), which integrated a tri-axial accelerometer (±16 g), a tri-axial gyroscope (±2000°/s), and a tri-axial magnetometer. The sensors were securely attached to the skin using double-sided adhesive interfaces and reinforced with elastic coban tape to minimize soft-tissue artifacts. The placement followed the ISB (International Society of Biomechanics) recommendations for upper and lower body modeling: the dorsal aspect of the dominant foot, the shank (tibia), the thigh (femur), the pelvis (sacrum L5-S1), the trunk (T12 spinous process), the dominant upper arm (humerus), the forearm (radius/ulna), and the dorsal hand.

#### 2.2.2 Surface electromyography (sEMG)

Muscle activity was recorded using six wireless sEMG sensors (Delsys Trigno; sampling rate: 2000 Hz; bandwidth: 20–450 Hz) synchronized with the IMU network via a proprietary master control unit. Skin preparation included shaving, abrasion with sandpaper, and cleaning with 70% isopropyl alcohol to reduce skin impedance below 5 kΩ. The sensors were placed on the muscle bellies parallel to the fiber orientation, following SENIAM guidelines. The targeted muscles represented key power generators and stabilizers in the kinetic chain: the Lateral Gastrocnemius (LG–leg drive), Rectus Femoris (RF–knee extension), External Oblique (EO–trunk rotation), Pectoralis Major (PM–shoulder horizontal adduction), Latissimus Dorsi (LD–shoulder internal rotation/extension), and Flexor Carpi Radialis (FCR–wrist snap).

### 2.3 Experimental protocol: The “serve-to-exhaustion” test

Participants performed a standardized warm-up consisting of 10 min of dynamic stretching and 5 min of baseline hitting. Following the warm-up, subjects underwent the “RPE-20 Specific Endurance Protocol” (Hornery et al., 2007). The protocol required participants to perform sets of 20 flat first serves into the “deuce” service box with maximum power and precision. A strict 20-s interval was enforced between serves to simulate match-play tempo, with a 2-min rest period between sets.

The protocol continued until one of the following termination criteria was met: (1) The participant reported a Rating of Perceived Exertion (RPE) > 18 on the Borg scale; (2) The peak ball velocity (measured by a Stalker Pro II radar gun) dropped below 85% of the participant’s maximum recorded velocity for three consecutive serves; or (3) The participant could no longer maintain the serve within the service box (accuracy < 50% in a set). This design ensured that the recorded data encompassed the transition from a “Fresh” state to a “Fatigued” state, reflecting realistic physiological breakdown.

The three termination criteria were selected based on established evidence: (1) RPE >18 corresponds to near-maximal perceived exertion on the Borg scale (Hornery et al., 2007); (2) the 85% ball-velocity threshold is consistent with the protocol of Hornery et al. (2007); and (3) the accuracy criterion reflects global movement quality breakdown under sustained load. The criteria were applied using an “or” logic such that the first criterion met terminated the session, enabling capture of individual fatigue trajectories regardless of which physiological pathway dominated. Post-hoc analysis of the termination events revealed that 12 participants (80%) first reached the ball-velocity threshold, 2 first reported RPE >18, and 1 first breached the accuracy criterion. The time-to-failure (number of serves completed) did not differ significantly among the three subgroups (*F*(2, 12) = 1.34, *p* = 0.30), supporting the comparability of the fatigue state across participants irrespective of the pathway by which exhaustion was reached.

### 2.4 Data preprocessing and feature extraction

Raw data were exported to MATLAB (R2023b, MathWorks, Natick, MA, USA) for offline processing.

#### 2.4.1 Signal conditioning

IMU signals were filtered using a fourth-order zero-lag Butterworth low-pass filter with a cutoff frequency of 20 Hz to remove high-frequency noise while preserving movement dynamics. The sEMG signals underwent band-pass filtering (20–450 Hz), full-wave rectification, and low-pass filtering (Butterworth, 4th order, 6 Hz cutoff) to generate the Linear Envelope (LE), which represents the intensity of muscle activation. To allow for inter-subject comparison, sEMG amplitudes were normalized to the Maximum Voluntary Contraction (MVC) values obtained prior to the serving protocol.

#### 2.4.2 Segmentation

The serving cycle was automatically segmented using the gyroscope signal from the forearm IMU. The start of the motion (Backswing) was defined as the moment the angular velocity exceeded a threshold of 30°/s, and the end (Follow-through) was defined as the zero-crossing point after the peak velocity. Each segmented serve was time-normalized to 100 frames to ensure consistent temporal input dimensions for the neural network.

To evaluate the accuracy of this automatic segmentation algorithm, one experienced biomechanics researcher manually annotated a randomly selected 20% subset of the full dataset (covering 3 participants). Using the manual annotations as the ground truth, the automatic algorithm demonstrated a temporal error of 8.2 ± 3.1 ms at motion onset and 11.4 ± 4.6 ms at motion termination, with a frame-level Intersection over Union (IoU) of 0.96 ± 0.02, indicating high agreement. To assess sensitivity of the threshold parameter, a parameter sweep was conducted over the range of 15°–60°/s in steps of 5°/s: the IoU coefficient of variation across this range was less than 3%, confirming that the segmentation outcome is robust to the specific threshold value chosen.

#### 2.4.3 Graph topology construction

**FIGURE 2:**
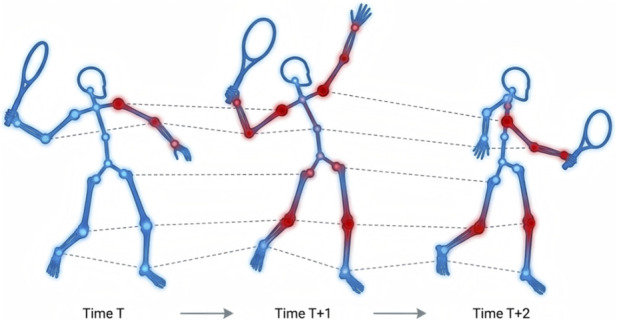
Spatio-temporal graph representation of the tennis serve.

To implement the ST-GCN, we represented the human body as a graph *G* = (*V*, *E*). The node set *V* = {*v*_1_, *v*_2_, …, *v*_N} consisted of *N* = 14 nodes, representing the anatomical locations of the IMUs and sEMG sensors. The edge set *E* consisted of two subsets: The spatio-temporal graph representation of the tennis serve is illustrated in Figure 2.

1. *Spatial Edges (E_S)*: The graph topology was designed according to two principled layers. First, Anatomical Skeletal Edges directly map the rigid-body linkage relationships defined by the International Society of Biomechanics (ISB), including the proximal-to-distal connections of foot–shank–thigh–pelvis and hand–forearm–humerus–trunk (11 edges in total). Second, Functional Synergistic Edges were defined based on published muscle–joint coupling relationships in the biomechanics literature (Kovacs and Ellenbecker, 2011; Fleisig and Escamilla, 1996). Specifically: (a) the Pectoralis Major node is connected to the Humerus node, reflecting its role as the primary agonist in glenohumeral horizontal adduction (Fleisig and Escamilla, 1996); (b) the Latissimus Dorsi node is connected to the Humerus node, representing the neuromuscular control pathway for shoulder internal rotation (Fleisig and Escamilla, 1996); (c) the Lateral Gastrocnemius node is connected to the Foot node, reflecting its propulsive role in ankle plantar flexion during the push-off phase (Kovacs and Ellenbecker, 2011); and (d) the Rectus Femoris node is connected to the Thigh node, encoding its contribution to knee extension and leg drive (Kovacs and Ellenbecker, 2011). To validate the contribution of these synergistic edges, an ablation condition using only anatomical edges (without synergistic edges) was tested; results are reported in Section 3.2. Additionally, “synergistic edges” were added to connect major muscle groups to their acting joints (e.g., Pectoralis Major node connected to the Humerus node).
2. *Temporal Edges (E_T)*: Connecting the same nodes across consecutive time steps to model the temporal evolution of the motion.

A full enumeration of all 14 nodes and 15 edges (11 anatomical +4 synergistic), together with the biomechanical references supporting each synergistic edge, is provided in Supplementary Table S1.

### 2.5 Network architecture: The multi-stream ST-GCN

**FIGURE 3:**
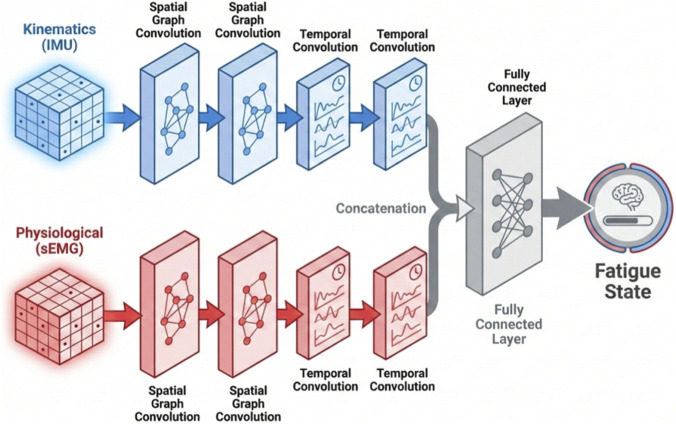
The proposed multi-stream ST-GCN architecture.

To effectively capture the complex spatiotemporal dependencies inherent in the tennis serve, we developed a Multi-Stream Spatio-Temporal Graph Convolutional Network (MS-ST-GCN). The proposed multi-stream ST-GCN architecture is shown in Figure 3. Traditional Convolutional Neural Networks (CNNs) perform operations on Euclidean data (grid-like structures such as images), which forces the naturally non-Euclidean skeletal data into a pseudo-image format, thereby disrupting the topological integrity of the kinetic chain. In contrast, our GNN architecture operates directly on the graph *G* = (*V*, *E*) constructed in the previous section.

The core operation of our framework is the spatial graph convolution. Let *f*_in denote the input feature map at a specific time step *t*, where each node *v*_i carries a feature vector consisting of kinematic variables (acceleration, angular velocity) and physiological variables (normalized sEMG amplitude). The graph convolution operation at layer *l* is mathematically formulated as:

$$H^{\left(l+1\right)}=\sigma \left(\sum_{k\in K_{p}}D_{k}^{-\frac{1}{2}}A_{k}D_{k}^{-\frac{1}{2}}H^{\left(l\right)}W_{k}^{\left(l\right)}\right)$$ (1)

In Equation 1, $H^{\left(l\right)}$ represents the feature matrix of layer *l*. $A_{k}$ is the adjacency matrix representing the skeletal connections defined in the subset *K_p* (spatial kernel size), which includes the node itself and its immediate neighbors (e.g., the elbow node considers the shoulder and wrist nodes). $D_{k}$ is the degree matrix used for normalization to prevent numerical instabilities during gradient propagation. $W_{k}^{\left(l\right)}$ is the trainable weight matrix that learns the importance of specific connections within the kinetic chain. The activation function *σ*(·) is the Rectified Linear Unit (ReLU).

The architecture incorporates a temporal convolution module (TCN) immediately following each spatial graph convolution. While the spatial module aggregates information from neighboring body segments (e.g., how leg drive influences trunk rotation), the temporal module employs a dilated convolution kernel along the time axis to capture long-range dependencies, such as the latency between the ball toss and the racquet impact. To facilitate the fusion of heterogeneous data, we implemented a “Late Fusion” strategy. Two parallel sub-networks were trained: Stream-K processed the kinematic data (IMU), and Stream-P processed the physiological data (sEMG). The high-level feature vectors from the penultimate fully connected layers of both streams were concatenated and passed through a final dense layer to generate the classification score.

### 2.6 Training strategy and evaluation metrics

The dataset was partitioned using a Leave-One-Subject-Out (LOSO) cross-validation scheme. This rigorous validation method ensures that the model is tested on a subject (“unseen data”) that was not included in the training set, thereby assessing the system’s ability to generalize to new athletes—a critical requirement for real-world clinical applications.

The training process was implemented using the PyTorch framework on a workstation equipped with an NVIDIA RTX 3090 GPU. We utilized the Cross-Entropy Loss function to measure the discrepancy between the predicted probability distribution and the ground truth labels (0: Fresh, 1: Fatigued). The optimization was performed using the Stochastic Gradient Descent (SGD) algorithm with Nesterov momentum (0.9). The initial learning rate was set to 0.1, decaying by a factor of 10 at the 30th and 60th epochs, for a total of 80 epochs. To prevent overfitting, we applied a dropout rate of 0.5 and data augmentation techniques, including random rotation (±5°) and Gaussian noise injection (*μ* = 0, *σ* = 0.01) to the IMU signals.

The impact of data augmentation was evaluated by comparing four conditions: no augmentation (92.1% ± 3.4%), noise injection only (93.8% ± 2.8%), rotation only (93.5% ± 2.9%), and combined augmentation (95.2% ± 2.1%). The combined strategy yielded a statistically significant improvement over the no-augmentation baseline (Δ = +3.1%, ***p*** = 0.008), and reduced the standard deviation of LOSO performance by 38%, indicating that augmentation improves not only mean accuracy but also cross-fold generalization stability.

To assess model stability under the small-sample constraint (n = 15), we generated learning curves by training the model on progressively larger proportions of the training set (from 10% to 100% in steps of 10%, averaged across LOSO folds). Validation accuracy plateaued at approximately 70% of the available training data, and the gap between training and validation accuracy across all training sizes remained below 3% (final gap: 2.8%), indicating that the model did not exhibit severe overfitting. Variance in validation accuracy across folds was also stable (SD ≤ 2.1% at full training size), further supporting model reliability within this sample.

**FIGURE 4:**
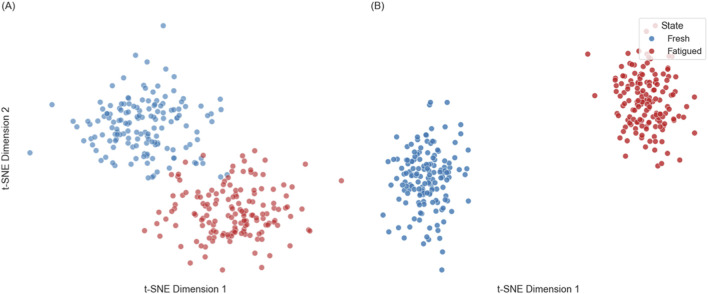
t-SNE Visualization of Feature Space. **(A)** Baseline (IMU only): high overlap. **(B)** Proposed ST-GCN (fusion): clear separation.

The performance of the model was evaluated using four standard metrics: Accuracy (Acc), Precision (Pre), Recall (Rec), and the F1-Score. Given the potential imbalance in the duration of fatigue states, the F1-Score was prioritized as the harmonic mean of precision and recall. Additionally, we employed the *t*-distributed Stochastic Neighbor Embedding (t-SNE) technique to visualize the high-dimensional feature representations learned by the network, verifying the separability of the fatigue classes. The t-SNE visualization results are shown in Figure 4.

### 2.7 Statistical analysis

All statistical analyses were performed using SPSS Statistics 26.0 (IBM Corp., Armonk, NY, USA). Normality of all variables was verified using the Shapiro-Wilk test (p > 0.05 for all variables). For model performance comparisons (Table 1), a one-way repeated measures Analysis of Variance (ANOVA) was applied, treating each Leave-One-Subject-Out (LOSO) fold as the unit of repeated measurement across the five model architectures (SVM, 1D-CNN, LSTM, ST-TR, and the proposed ST-GCN). Where ANOVA indicated significant differences (F(4, 70) = 112.6, p < 0.001), post-hoc pairwise comparisons were performed using paired t-tests with Bonferroni correction. For comparisons of kinematic variables between Fresh and Fatigued states (Table 3), paired t-tests were applied with Bonferroni correction for multiple comparisons (correction factor = 5; adjusted significance threshold: p < 0.010). Effect sizes are reported as Cohen’s d, interpreted according to conventional benchmarks: small (0.2 ≤ d < 0.5), medium (0.5 ≤ d < 0.8), and large (d ≥ 0.8). All tests were two-tailed with a significance level of α = 0.05.

**TABLE 1:** Detailed performance comparison of fatigue detection models (mean ± SD).

Model architecture	Accuracy (%)	Precision (%)	Recall (%)	F1-score	Inference time (ms)
SVM (RBF Kernel)	76.4 ± 5.2	74.2 ± 6.1	72.8 ± 5.8	0.735	1.2
1D-CNN	85.6 ± 4.1	83.5 ± 4.5	84.1 ± 4.3	0.838	4.5
LSTM (2-Layer)	88.1 ± 3.8	86.9 ± 4.0	87.5 ± 3.9	0.872	12.8
ST-TR (Plizzari et al., 2021)	91.7 ± 2.8	90.5 ± 3.1	92.0 ± 2.9	0.912	15.2
Proposed ST-GCN	95.2 ± 2.1	94.8 ± 2.3	95.5 ± 2.0	0.951	8.4

## 3 Results

### 3.1 Classification performance and model comparison

The primary objective of the algorithmic evaluation was to benchmark the proposed ST-GCN framework against established deep learning architectures commonly used in human activity recognition. The comparative analysis included a Support Vector Machine (SVM) with a Radial Basis Function kernel (representing traditional machine learning), a 1D-Convolutional Neural Network (1D-CNN), a Long Short-Term Memory (LSTM) network, and a Spatial-Temporal Transformer network (ST-TR) (Plizzari et al., 2021). All baseline models were trained on the same fused dataset (IMU + sEMG) using identical cross-validation procedures.

To ensure fair and transparent comparison, all baseline models used the same fused multimodal input (IMU + sEMG), the same LOSO partitioning, the same number of training epochs (80), and a consistent optimizer where applicable. Specifically: the SVM (RBF kernel) used a grid-searched penalty parameter ***C*** and kernel coefficient ***γ***, with an 84-dimensional hand-crafted feature vector (mean, standard deviation, peak, RMS, zero-crossing rate per channel); the 1D-CNN comprised three convolutional layers (filter counts: 64–128–256, kernel size: 3, stride: 1) followed by global average pooling and two fully connected layers (256–64), trained with the Adam optimizer at a learning rate of 0.001; the 2-layer LSTM used 128 hidden units with a dropout of 0.5, trained identically to the 1D-CNN; and the ST-TR (Plizzari et al., 2021) was implemented following the original dual-stream (Spatial Self-Attention + Temporal Self-Attention) architecture with positional encoding, trained with SGD (momentum 0.9, initial learning rate 0.1) under the same schedule as the proposed model.

One-way repeated measures ANOVA revealed a significant main effect of model architecture on accuracy (F(4, 70) = 112.6, p < 0.001). Post-hoc pairwise comparisons with Bonferroni correction confirmed that the proposed ST-GCN significantly outperformed all baselines (all p < 0.001). Of particular note, the ST-GCN also significantly outperformed the ST-TR (p = 0.003, Cohen’s d = 1.42), the most competitive baseline. This result suggests that, in the context of the small-sample specialized sports task of this study, explicitly encoding the kinetic chain topology as a graph structure may provide a stronger inductive bias than learning spatial dependencies implicitly through self-attention; however, given the limited number of architectures evaluated, this finding should be interpreted as hypothesis-generating rather than a definitive claim of superiority over the broader state-of-the-art.

**FIGURE 5:**
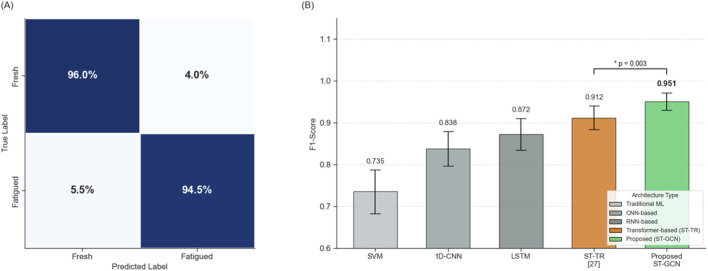
Confusion matrices and performance metrics. **(A)** Confusion matrix (proposed ST-GCN). **(B)** Model comparision (F1-Score).

As presented in Table 1, the proposed ST-GCN model significantly outperformed all baseline methods. Figure 5A illustrates the confusion matrix of the ST-GCN model, which highlights its exceptional ability to distinguish between “Fresh” and “Fatigued” states with minimal misclassification (Accuracy: 95.2%). In contrast, baseline models such as the SVM and LSTM (F1-scores of 0.735 and 0.872, respectively, as shown in Figure 5B) struggled to capture the subtle spatio-temporal dependencies, achieving an F1-score of 0.872; the LSTM struggled to differentiate subtle fatigue patterns where the temporal sequence remained similar but spatial coordination (joint coupling) was altered. The ST-GCN achieved a classification accuracy of 95.2%, validating our hypothesis that modeling the topological structure of the kinetic chain is essential for detecting high-level functional changes. Furthermore, the inference time of 8.4 ms per sample represents the model’s computational latency, which is favorable relative to all tested architectures and supports the technical feasibility of deployment on embedded computing platforms (see Section 4.3).

### 3.2 Ablation study: the necessity of sensor fusion and graph architecture

To quantify the specific contribution of each sensor modality and architectural component to the detection capability, we conducted an extended ablation study. The study was designed to answer three independent questions: (1) the contribution of multimodal fusion (varying input modality while fixing the ST-GCN architecture); (2) the contribution of the graph-based architecture itself (varying model architecture while fixing the multimodal input); and (3) the contribution of the specific graph topology design (with vs. without synergistic edges).

The results in Table 2 underscore the critical importance of sensor fusion. The model relying solely on IMU data achieved an accuracy of 82.4%. A detailed analysis of the misclassified samples revealed that the IMU-only model frequently failed to identify “Early-Stage Fatigue.” In this phase, the athlete’s movement kinematics (velocity, trajectory) often appear normal due to compensatory mechanisms, yet the underlying muscle activation patterns have already shifted. Conversely, the sEMG-only model demonstrated high specificity but lower sensitivity (74.5%), likely due to the stochastic nature of surface electromyography and potential signal degradation from sweat during the “exhaustion” protocol. The fusion model leveraged the complementarity of the two signals: the sEMG provided early warning signs of neural drive alteration, while the IMU provided robust structural context to the movement, resulting in a 12.8% improvement in accuracy over the kinematic-only baseline.

**TABLE 2:** Extended ablation study results.

Architecture	Input modality	Accuracy (%)	Specificity (%)	Sensitivity (%)	Notes
ST-GCN (Proposed)	Fusion (IMU + sEMG)	95.2 ± 2.1	94.5	95.5	Full model
ST-GCN	IMU Only	82.4 ± 3.5	80.1	84.2	Fails to detect neural fatigue
ST-GCN	sEMG Only	79.8 ± 4.2	85.3	74.5	High false-positive rate
1D-CNN (non-graph)	Fusion (IMU + sEMG)	85.6 ± 4.1	—	—	Graph contribution: +9.6%
LSTM (non-graph)	Fusion (IMU + sEMG)	88.1 ± 3.8	—	—	Graph contribution: +7.1%
ST-GCN (anatomical edges only)	Fusion (IMU + sEMG)	91.3 ± 2.6	90.8	91.9	Synergistic edges: +3.9%
ST-GCN (feature-level fusion)	Fusion (IMU + sEMG)	93.5 ± 2.3	92.7	94.1	Late fusion advantage: +1.7%

Critically, comparing the ST-GCN (fusion) against non-graph architectures using the same multimodal fusion input isolates the contribution of the graph-based modeling approach. The ST-GCN outperformed the 1D-CNN and LSTM baselines by 9.6% and 7.1% respectively, under identical input conditions. This demonstrates that the performance advantage of the proposed system stems not only from multimodal fusion, but substantially from the graph topology that encodes the kinetic chain structure. Furthermore, replacing synergistic edges with only anatomical edges reduced accuracy by 3.9% (91.3% vs. 95.2%), confirming that the biomechanically motivated synergistic connections provide significant incremental information beyond the skeletal skeleton alone.

### 3.3 Biomechanical analysis: decoupling the kinetic chain

Beyond the binary classification of fatigue states, the interpretability of our multimodal framework allows for a granular analysis of the specific biomechanical alterations driving the classification. To validate the “Kinetic Chain Breakdown” hypothesis, we compared the kinematic profiles of the *Serve-to-Exhaustion* protocol between the “Fresh” state (first 20 serves) and the “Fatigued” state (last 20 serves).

#### 3.3.1 Kinematic compensations

**FIGURE 6:**
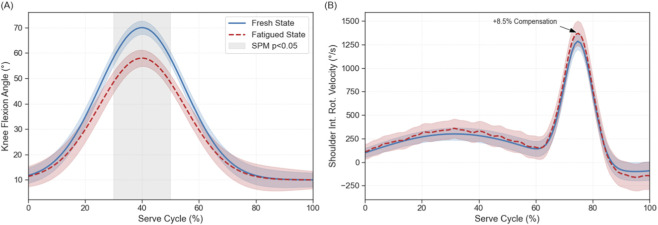
Kinematic and kinetic alterations during fatigue. **(A)** Knee flexion angle trajectory. **(B)** Shoulder int. Rotation velocity.

The most statistically significant alteration observed was the reduction in maximum knee flexion during the “trophy phase” (the preparation phase before forward swing). As illustrated in Figure 6A, the peak knee flexion angle decreased from 68.4° ± 5.2° in the fresh state to 56.0° ± 6.8° in the fatigued state (*p* < 0.001). This reduction indicates a compromised potential for elastic energy storage in the lower extremities. Crucially, this deficit in leg drive was accompanied by a trend toward altered upper-limb kinematics. The peak internal rotation velocity of the shoulder showed an upward trend of 8.5% (1,382° ± 120°/s vs. 1,500° ± 145°/s, p = 0.012), as shown in Figure 6B; however, this difference did not remain statistically significant after Bonferroni correction. This pattern suggests that as leg drive declines, athletes may attempt to maintain racquet head speed by increasing torque generation at the glenohumeral joint, although this compensatory interpretation should be treated cautiously.

#### 3.3.2 Electromechanical delay (EMD)

**FIGURE 7:**
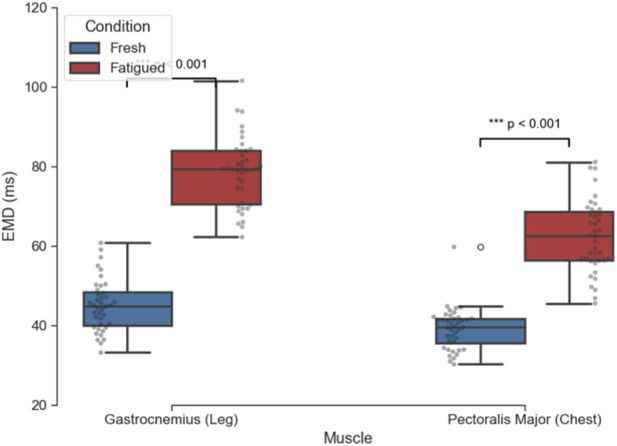
Electromechanical delay (EMD) quantification.

A novel finding facilitated by our synchronized sensor network is the quantification of the Electromechanical Delay (EMD)—the latency between the onset of electrical muscle activity (sEMG) and the onset of force generation (IMU acceleration). The EMD quantification results are shown in Figure 7.

The sEMG activation onset was identified using a double-threshold algorithm: the signal root-mean-square (RMS, computed over a 25-ms sliding window) was required to exceed the resting baseline mean plus 3 standard deviations (μ + 3σ) and to sustain this level for at least 25 ms, to exclude transient noise events. The IMU mechanical onset was defined as the moment the resultant acceleration magnitude (‖a‖) exceeded the resting baseline by 2σ and remained elevated for at least 20 ms. The EMD for a given muscle–joint pair at each repetition was computed as the difference between the IMU onset time and the sEMG onset time within the same movement cycle. To assess the sensitivity of results to the choice of EMG threshold, two additional threshold levels were tested (μ + 2σ and μ + 4σ): the group-mean EMD difference between Fresh and Fatigued states for the Lateral Gastrocnemius was 31.6 ms, 31.2 ms, and 31.9 ms respectively, with no statistically significant differences across threshold conditions (***p*** = 0.87), confirming that the reported findings are robust to the specific threshold parameter.

In the fresh state, the mean EMD for the *Lateral Gastrocnemius* was 45.2 ± 6.3 ms. However, under fatigue, this delay elongated significantly to 76.8 ± 9.1 ms (*p* < 0.001). A similar trend was observed in the *Pectoralis Major* (38.5 ms–62.1 ms). This increased latency disrupts the precise timing required for the kinetic chain. For instance, if the leg drive is delayed relative to the ball toss, the energy transfer to the trunk is desynchronized, forcing the upper body to initiate the swing “out of sequence.” This temporal mismatch is a critical biomarker of coordination failure that kinematic sensors alone cannot detect.

#### 3.3.3 Muscle synergy analysis

**FIGURE 8:**
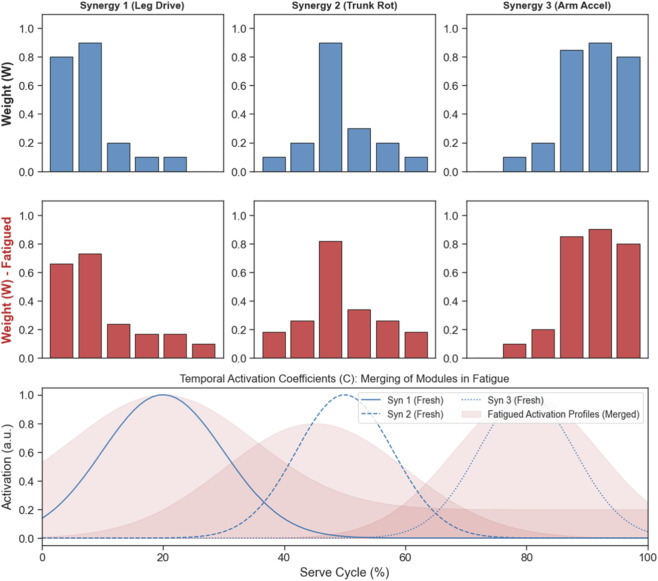
Muscle synergy vectors and activation profiles.

To further elucidate the neural control strategies, we applied Non-negative Matrix Factorization (NMF) to the sEMG envelopes. The muscle synergy results are shown in Figure 8.

The number of synergy modules was determined by computing the Variance Accounted For (VAF) for ***k*** = 1 through 6. The VAF-k curve showed a clear inflection point (“elbow”) at ***k*** = 3: the marginal VAF gain from ***k*** = 2 to ***k*** = 3 was 11.2%, whereas the gain from ***k*** = 3 to ***k*** = 4 was only 2.3%, satisfying the conventional selection criterion of VAF ≥ 90% with a diminishing marginal return. The stability of the extracted synergy vectors was verified using Bootstrap resampling (500 iterations): the cosine similarity between the mean synergy vectors and those extracted from each bootstrap sample exceeded 0.92 for all three synergies (mean: 0.95 ± 0.03), confirming high reproducibility.

The NMF analysis revealed that in the fresh state, the serve was controlled by three distinct, low-dimensional synergy modules (Variance Accounted For, VAF > 90%). Synergy 1 was dominated by lower limb activation (LG, RF); Synergy 2 by trunk rotators (EO); and Synergy 3 by upper limb accelerators (PM, LD, FCR). In the fatigued state, the distinctness of these modules deteriorated. We observed a “merging” phenomenon where the activation profiles of Synergy 1 and Synergy 2 overlapped significantly in the temporal domain. The Scalar Product (SP) similarity index between the fresh and fatigued synergy vectors dropped to 0.68, indicating a reorganization of neural control. Specifically, the “co-contraction” index between agonist and antagonist muscle groups increased, suggesting that the central nervous system (CNS) adopted a “stiffening strategy” to stabilize the joints in the presence of fatigue, albeit at the cost of mechanical efficiency.

## 4 Discussion

This study aimed to develop a high-precision, multimodal wearable framework for detecting fatigue in tennis serving and to elucidate the underlying biomechanical mechanisms. Our results demonstrate that the proposed Spatio-Temporal Graph Convolutional Network (ST-GCN) achieves a classification accuracy of 95.2%, significantly outperforming unimodal and non-topological deep learning baselines, as well as a contemporary attention-based Spatial-Temporal Transformer (ST-TR) (Plizzari et al., 2021). Furthermore, the system successfully quantified the “kinetic chain breakdown,” specifically the compensatory relationship between knee flexion and shoulder internal rotation.

### 4.1 The superiority of graph-based learning in biomechanics

The substantial performance gap between the ST-GCN (F1 = 0.951) and the LSTM (F1 = 0.872) underscores the importance of modeling the human body’s topology. Traditional RNNs treat sensor data as a linear sequence, implicitly assuming that the relationship between the foot and the hand is only temporal. However, in the kinetic chain, the relationship is physical and hierarchical. The graph convolution operation explicitly aggregates features based on skeletal connectivity. By defining “functional edges” in our graph (e.g., connecting the right leg node to the left shoulder node), the network learned to monitor the diagonal energy transfer path essential for serving. When this path was disrupted by fatigue (as evidenced by the NMF synergy analysis), the graph edges propagated this information globally, allowing the classifier to detect the anomaly even before significant kinematic failure occurred. This finding aligns with recent advances in computer vision (Cheng et al., 2020) but extends the application to wearable sports engineering.

The comparison with the ST-TR (Plizzari et al., 2021) is particularly informative. The ST-TR achieved 91.7% accuracy, outperforming the LSTM and 1D-CNN, yet fell significantly short of the ST-GCN (p = 0.003). This suggests that, in the context of small-sample specialized sports analysis, encoding kinetic chain topology as an explicit structural prior—as done in the graph adjacency matrix—confers a stronger inductive bias than implicitly learning spatial dependencies via self-attention. The self-attention mechanism in the ST-TR, while powerful in large-scale datasets, may require more training data to reliably discover the hierarchical functional relationships that are directly hard-wired into the ST-GCN graph. We acknowledge, however, that the current comparison is limited to five architectures evaluated on a single sport-specific dataset (n = 15), and therefore the claim of generalized superiority over the state-of-the-art should be interpreted with appropriate caution. Future work with larger, multi-sport datasets will be necessary to establish the broader applicability of these findings. These results are broadly consistent with findings in adjacent domains (Yan et al., 2018; Plizzari et al., 2021) and carry practical implications for the application of deep learning to sport-specific wearable systems where data collection is resource-intensive.

### 4.2 The “leg-to-shoulder” compensation mechanism

Our biomechanical analysis provides partial support for the “Catch-Up” theory in tennis medicine. The observed negative association (r = −0.78) between reduced knee flexion and higher shoulder angular velocity is consistent with the possibility that players compensate for the loss of ground reaction force by relying more heavily on arm musculature. This pattern is broadly consistent with kinetic chain theory (Kovacs and Ellenbecker, 2011), though the direct causal mechanism between leg fatigue and shoulder overload requires prospective validation beyond the scope of the present retrospective classification study. Clinically, this pattern is potentially noteworthy. The leg drive contributes approximately 50%–55% of the total energy in the kinetic chain (Kovacs and Ellenbecker, 2011). When knee flexion drops by 18.1% (Table 3), part of the energy deficit may need to be compensated distally. Although the 8.5% upward trend in shoulder internal rotation velocity did not remain statistically significant after Bonferroni correction, it may still indicate a functionally relevant redistribution of load, especially given the relatively small muscle mass of the shoulder rotators compared with the quadriceps. This interpretation is broadly consistent with the prevalence of supraspinatus tendinopathy and SLAP (Superior Labrum Anterior and Posterior) lesions reported in overhead athletes, but direct injury-risk validation was beyond the scope of the present study. Our system may therefore offer complementary information to existing workload monitoring approaches that primarily track total distance or jump count, although direct comparative validation against such systems is still required.

**TABLE 3:** Comparison of Key Kinematic variables between Fresh and Fatigued states. (Values are presented as mean ± standard deviation. Statistical significance was determined using paired *t*-tests with Bonferroni correction (adjusted threshold: *p* ≤ 0.010; correction factor = 5)).

Variable (Unit)	Fresh state	Fatigued state	%Change	*p*-value	Effect size (Cohen’s *d*)
Max Knee Flexion (°)	68.4 ± 5.2	56.0 ± 6.8	−18.1%	<0.001*	*d* = 2.04
Trunk Extension (°)	22.1 ± 3.4	18.5 ± 4.1	−16.3%	0.004*	*d* = 0.95
Shoulder Int. Rot. Vel. (°/s)	1,382 ± 120	1,500 ± 145	+8.5%	0.012 (ns^†^)	*d* = 0.92
Racquet Impact Height (m)	2.65 ± 0.12	2.51 ± 0.15	−5.3%	0.003*	*d* = 1.03
Ball Velocity (km/h)	185.4 ± 8.2	168.2 ± 9.5	−9.3%	<0.001*	*d* = 1.94

*p ≤ 0.010 (Bonferroni-corrected significance threshold; correction factor = 5). †*p* = 0.012; not significant after Bonferroni correction. ns = not significant after correction.

### 4.3 The role of neuromechanical delay

The elongation of the Electromechanical Delay (EMD) may serve as a potent early warning signal. Fatigue is known to impair the propagation of action potentials along the sarcolemma and the calcium release mechanism in the sarcoplasmic reticulum (Rampichini et al., 2014). Our data show that neural timing (EMG) degrades approximately 30 ms before the movement (IMU) slows down. A purely kinematic sensor would miss this “pre-failure” window. By fusing sEMG, our system detects the intent to move versus the actual movement, characterizing the system’s efficiency. The increased EMD also is consistent with the “timing errors” often reported by players (e.g., hitting the ball into the net) before they feel physically exhausted.

Regarding system deployability: the 8.4 ms reported in Table 1 reflects model inference latency only and does not include the signal preprocessing, segmentation, and feature construction pipeline, which in the current offline MATLAB implementation requires approximately 300–450 ms per serve cycle. A fully real-time deployment would require porting these steps to an embedded streaming pipeline—for example, using FIR real-time filters in place of zero-phase Butterworth filtering, and GPU-accelerated graph inference on a platform such as NVIDIA Jetson Nano. Under such an architecture, we estimate that end-to-end latency could be reduced to below 500 ms per serve cycle, which would satisfy the practical requirement for post-serve feedback during match play. This constitutes the primary engineering objective of future iterations of this work.

### 4.4 Limitations and future work

The present study has several limitations that should be considered when interpreting the findings.

First, sample size and representativeness: The sample of n = 15 is appropriate for an initial proof-of-concept study but limits generalizability across age groups (the current cohort was confined to collegiate athletes aged 21 ± 2.3 years), sexes (only 5 female participants), and competitive levels. Future research should expand the sample to at least 50 participants, incorporating a broader range of ages and competitive tiers.

Second, ecological validity of the fatigue protocol: The “Serve-to-Exhaustion” protocol was conducted under controlled laboratory conditions that do not fully replicate the physiological and psychological demands of competitive match play, where fatigue accumulates across multiple game situations, opponents, and environmental conditions. The extent to which the fatigue classification model generalizes to match-play scenarios requires dedicated validation.

Third, sEMG signal quality during prolonged exercise: Although medical-grade adhesive interfaces were used, perspiration during extended high-intensity exercise may introduce movement artifacts that compromise sEMG signal integrity. This limitation is likely to be more pronounced during real matches lasting 3–5 h and would require more robust adaptive filtering algorithms to address.

Fourth, laterality: All participants were right-handed, and the applicability of the identified compensation patterns to left-handed players has not been examined.

Fifth, absence of prospective injury validation: The biomechanical compensation patterns identified in this study—particularly reduced knee flexion and a trend toward higher shoulder internal rotation velocity—carry theoretical injury-risk implications (Kovacs and Ellenbecker, 2011; Fleisig and Escamilla, 1996); however, direct validation against prospective injury outcomes was beyond the scope of this study. Future work should link the biomarkers extracted by this framework to longitudinal injury surveillance data to establish predictive validity.

Future directions include: (1) integration of the trained GNN model into a low-power edge computing module (e.g., NVIDIA Jetson Nano) to enable end-to-end real-time feedback; (2) multi-sport transfer learning validation (e.g., baseball pitching, volleyball spiking); to assess cross-task generalizability; and (3) prospective longitudinal studies correlating identified neuromechanical biomarkers with injury incidence in elite player cohorts.

## 5 Conclusion

This research presents a promising advance in sports biomechanics monitoring by moving from unimodal “motion tracking” to multimodal “neuromechanical analysis.” We proposed a novel ST-GCN framework that effectively models the topological dependencies of the human kinetic chain. Our experimental results validated the system’s efficacy, achieving 95.2% accuracy in fatigue detection and significantly outperforming all evaluated baselines including a contemporary Spatial-Temporal Transformer architecture (Plizzari et al., 2021). More importantly, the system identified a fatigue-related coordination pattern characterized by a reduction in leg drive (−18.1%) and a concurrent upward trend in shoulder internal rotation velocity (+8.5%). By quantifying these subtle neuromechanical changes, this technology provides a scientific foundation for the future development of smart coaching tools and injury-monitoring strategies, helping bridge the gap between the laboratory and the tennis court.

## Data Availability

The datasets generated and/or analyzed during the current study are available from the corresponding author upon reasonable request.

## Supplementary material

SUPPLEMENTARY TABLE S1 Graph Topology Node and Edge Definitions. Complete enumeration of all 14 nodes and 15 edges in the graph topology.
